# An Early-Stage Digital Therapeutic Intervention to Enhance Affective Response During Physical Activity Among Adults With Overweight or Obesity: Benchmark-Driven Formative Testing Study

**DOI:** 10.2196/71472

**Published:** 2026-02-20

**Authors:** Rachel Crosley-Lyons, Lori Hatzinger, Micaela Hewus, Wei-Lin Wang, Delfien Van Dyck, Jimi Huh, Eric Hekler, Genevieve F Dunton

**Affiliations:** 1 Department of Population and Public Health Sciences Keck School of Medicine University of Southern California Los Angeles, CA United States; 2 Department of Movement and Sports Sciences Ghent University Ghent Belgium; 3 Herbert Wertheim School of Public Health and Human Longevity Science University of California, San Diego San Diego, CA United States; 4 Center for Wireless & Population Health Systems University of California, San Diego San Diego, CA United States; 5 Department of Psychology University of Southern California Los Angeles, CA United States

**Keywords:** digital therapeutics, real world evidence, formative study, MOST framework, SMART goals, mHealth, physical activity, affective response

## Abstract

**Background:**

Mobile device–enabled interventions known as digital therapeutics (DTx) are increasingly used to prevent chronic disease by targeting psychological and behavioral processes. Individuals’ unique experiences while receiving DTx comprise real-world evidence (RWE) for evaluating DTx performance. An emerging strategy for early-stage DTx formative work uses small sample sizes, which facilitate efficient iteration and agile learning, while evaluating performance against descriptive benchmarks defined a priori, therefore minimizing the risk for confirmation bias. This study test benchmarks from the DTx RWE framework to formatively evaluate a novel DTx (the eMOTION study) to enhance affective response (ie, how people feel) during physical activity (PA).

**Objective:**

This study aimed to determine whether the eMOTION DTx met a priori benchmarks for safety (<1% of participants report an adverse event), plausibility (≥51% of participants experience increased enjoyment in PA), usability (eg, ≥51% of participants report adequate usability), sustainability, feasibility (eg, <70% of participants report dissatisfaction), and equity (equity and accessibility are approximately equal across subgroups).

**Methods:**

Participants (N=36; mean age 46, SD 14 years; 20/37, 54% female) underwent stratified random assignment to test one of four DTx versions for 14 days (n=9 each): (1) intensity PA goals, (2) affect PA goals with type and context recommendations, (3) affect PA goals with savoring exercises, and (4) affect PA goals with type, context, and savoring. Participants completed daily intervention sessions, asking them to focus on achieving a target heart rate (intensity) or feeling good (affect) during PA. Smartwatches were used to track PA and answer ecological momentary assessment (EMA) questions about how they felt during PA. Performance toward benchmarks was primarily assessed via official Institutional Review Board reporting channels (safety), interviews (plausibility, accessibility, and usability), and questionnaires (System Usability Scale [usability], Delighted-Terrible Scale [sustainability and feasibility], and equity).

**Results:**

The eMOTION DTx versions exceeded all a priori safety, plausibility, accessibility, usability, sustainability, feasibility, and equity thresholds. For safety, no adverse events were reported. Regarding plausibility, more than half of the participants who received affect goals reported increased PA enjoyment at the end of the study. Moreover, 64%-72% (23-26 out of 36) of participants rated the DTx at or above the standard System Usability Scale cutoff point for acceptable usability. More than 60% (22/36) of participants reported satisfaction with all DTx components, supporting DTx sustainability and feasibility. Finally, there was evidence for equity, with plausibility and accessibility comparable across sex, race, ethnicity, income, age, BMI, mobility, and physical constraint subgroups.

**Conclusions:**

Since DTx RWE Framework benchmarks for safety, plausibility, accessibility, usability, sustainability, feasibility, and equity were largely met, the eMOTION Study DTx is ready for a full-scale efficacy trial to refine the DTx and optimize efficiency and feasibility. Our approach incorporated transparent decision-making to generate results that are more readily translatable, easily replicable, and reflect current best practices in the field of DTx.

**Trial Registration:**

ClinicalTrials.gov NCT06125964; https://clinicaltrials.gov/study/NCT06125964

## Introduction

### Digital Therapeutics

Widespread smartphone ownership and expanded wearable device capabilities have steadily facilitated a paradigm shift in health care delivery. The medical system has traditionally used in-person examinations and pharmaceuticals to detect, treat, and manage chronic diseases. However, such diagnostic care is incredibly costly—with global spending projected to reach US $47 trillion by 2030—and inefficient, given that many noncommunicable diseases are potentially avoidable via behavior modification [1]. While clinicians counsel patients about the importance of a healthy lifestyle for mitigating chronic disease risk, compliance with preventive care recommendations remains low. Digital therapeutics (DTx)—mobile device software systems that deliver evidence-based behavioral intervention packages to treat or prevent disease in real-world environments—are poised to complement and improve upon these efforts. For example, DTx can extend preventive care beyond the clinic by facilitating behavior change in daily life and empowering patients to monitor their own progress. High-resolution digital treatment evaluation data collected by DTx serve as real-world evidence (RWE) that can be used for efficient performance assessments during research and development. Even as forecasts show a DTx market compound annual growth rate of 27.2% until 2030 [2], the transition from DTx research and development to regulatory testing and public release remains disjointed and inefficient.

### Benchmark-Driven Formative Testing as a Rigorous Approach to Early-Stage DTx Development

DTx involves complex sociotechnical systems with multiple interdependent components—user interfaces, behavioral protocols, technical infrastructure, and human-technology interactions—each of which must function adequately for the intervention to succeed. Failure to establish a robust foundation across all these components before initiating large-scale trials substantially increases the risk of type III error, wherein a study is conducted rigorously but fails to test what it intended to test because the intervention itself was inadequately developed or the control condition was poorly specified.

Traditional approaches to early-stage DTx research face a methodological paradox: formative studies must remain small to enable rapid, resource-efficient learning and iteration; yet, small sample sizes preclude the use of inferential statistics with adequate statistical power. This creates a problematic situation where researchers either (1) inappropriately apply underpowered inferential tests that are likely to produce unreliable results, or (2) rely solely on qualitative interpretation of descriptive results, which introduces substantial risk of confirmation bias, the well-documented human tendency to interpret ambiguous data in ways that confirm preexisting beliefs and expectations.

To address this methodological challenge, leading experts in behavioral intervention science and DTx development have advocated for benchmark-driven formative testing as a rigorous alternative approach. This methodology, articulated in frameworks such as the Obesity-Related Behavioral Intervention Trials (ORBIT) model for behavioral intervention development and Phase II of the DTx RWE Framework, establishes a priori performance thresholds that can be evaluated using purely descriptive statistics while maintaining scientific rigor [3].

### The Logic of Benchmark-Driven Formative Testing

Benchmark-driven formative testing addresses 4 fundamental tensions in early-stage intervention research.

First, the need for efficient learning requires small sample sizes that enable rapid iteration cycles and resource-efficient exploration of intervention components, implementation strategies, and potential adaptations for diverse populations. Large trials are essential for establishing generalizability, but they are inappropriate tools for the formative work of intervention refinement.

Second, the inappropriateness of underpowered inferential statistics in small samples is well established. When sample sizes are insufficient for adequate statistical power, *P* values become unreliable indicators of true effects, CIs become too wide to be informative, and the risk of both false positives and false negatives increases substantially. Running inferential statistics on samples of N=30-50 participants typically yields a power of 0.20-0.40 for detecting medium effect sizes—well below the conventional 0.80 standard. In such contexts, null hypothesis significance testing provides an illusion of rigor while actually undermining the validity of conclusions [4,5].

Third, the risk of confirmation bias affects all human judgment, including scientific interpretation. Without prespecified criteria for success, researchers may unconsciously interpret ambiguous results favorably, seeing “promising trends” or “encouraging patterns” that align with their hypotheses. This risk is particularly acute in small samples where data can appear to support multiple narratives depending on how one frames the analysis.

Fourth, the distinction between statistical and clinical significance is critical but often conflated. A statistically significant finding may have minimal real-world impact, while a clinically meaningful effect may not reach statistical significance in an underpowered study. Effect sizes, minimal clinically important differences (MCIDs), and practical significance must be considered alongside *P* values; yet, traditional null hypothesis testing frameworks prioritize statistical significance over substantive meaningfulness [6].

### How Benchmarks Address These Tensions

A priori establishment of benchmarks addresses all 4 tensions simultaneously.

#### Enabling Small, Efficient Samples

Because benchmarks can be evaluated descriptively (eg, “did 51% of participants report increased PA enjoyment?”). They do not require the large samples necessary for adequate statistical power. This enables formative studies to remain small enough for rapid iteration while still providing clear, interpretable evidence about intervention performance.

#### Avoiding Inappropriate Statistical Tests

Benchmark evaluation relies on descriptive statistics—percentages, frequencies, and comparisons to predetermined thresholds—rather than inferential tests. This eliminates the problems associated with underpowered hypothesis testing while still providing rigorous evaluation criteria relevant for formative work within small sample sizes.

#### Minimizing Confirmation Bias

By establishing success criteria before data collection, benchmark approaches create an objective standard against which results must be evaluated. If the benchmark specifies that ≥51% of participants must report increased PA enjoyment, then 45% represents clear failure to meet the criterion, regardless of how “close” it might feel or how researchers might be tempted to interpret it favorably. The threshold is predetermined and nonnegotiable.

#### Centering Clinical Meaningfulness

Benchmarks are explicitly designed to reflect what would constitute meaningful real-world impact, not merely statistical detectability. For example, in our study here, a 51% threshold for plausibility reflects the expectation that an intervention should benefit the majority of recipients—a clinically meaningful standard that is independent of sample size or statistical power considerations. Benchmarks can incorporate established MCIDs, align with clinically significant thresholds from previous literature, and balance what is both meaningful and plausibly achievable given the current state of the science.

#### Benchmark Selection and Justification

Rigorous benchmark-driven research requires that thresholds be established through a systematic process that balances clinical meaningfulness with plausible achievability based on previous evidence. Given the novelty of this line of work, our team developed benchmarks that have an initial plausibility of balancing both ambition and likelihood of being observed. While these benchmarks were not explicitly based on previous literature—due to the lack of extant work—they represent a potential baseline that others can improve upon in future proof-of-concept trials.

The process of benchmark development should be transparent and documented, ideally through preregistration, to ensure that thresholds are truly a priori rather than post hoc. In our study, benchmarks were established before data collection and documented in our preregistered analysis plan on Open Science Framework [7].

#### Interpreting Benchmark Results

Critically, results from benchmark-driven formative studies must not be interpreted as generalizable to broader populations. Meeting benchmarks indicates that an intervention has achieved a sufficient foundation to warrant progression to the next development phase, but it does not establish external validity or population-level efficacy or effectiveness. Those determinations require adequately powered randomized controlled trials with representative samples.

Instead, benchmark-driven formative testing serves a specific purpose in the intervention development pipeline: to establish whether all essential components of an intervention function adequately, whether the intervention can be delivered as intended, whether it demonstrates sufficient signal of benefit to justify further investment, and whether potential implementation barriers can be identified and addressed before committing to large-scale testing. This is analogous to Phase I/II trials in pharmaceutical development, which establish safety, dosing, and preliminary efficacy signals before proceeding to Phase III confirmatory trials.

Meeting formative benchmarks provides evidence that an intervention is “ready” for larger-scale testing—that the foundation is sufficiently solid that a properly powered trial can be conducted without high risk of Type III error. Failing to meet benchmarks indicates that further refinement is needed before proceeding, thereby protecting against the waste of resources on large trials of interventions that are not yet adequately developed.

#### Rigor in Formative Research

The methodological rigor of formative research is achieved not through the application of inferential statistics to underpowered samples, but through:

1. Systematic benchmark establishment grounded in previous literature and clinical meaningfulness.
2. Prespecification and transparent documentation of all benchmarks before data collection.
3. Comprehensive assessment across multiple domains (safety, plausibility, usability, feasibility, and equity).
4. Objective evaluation of whether benchmarks were met using descriptive statistics.
5. Honest interpretation that acknowledges the formative, nongeneralizable nature of findings.
6. Iterative refinement based on benchmark performance before proceeding to larger trials.

This approach represents a methodologically sound framework for early-stage intervention development that avoids the false precision of underpowered inferential testing while maintaining scientific rigor through prespecified, clinically meaningful evaluation criteria. As Czajkowski and colleagues [3] note in their comprehensive guide to behavioral intervention development, early-stage work should prioritize intervention optimization, rather than hypothesis testing—a fundamentally different scientific objective that requires different methodological tools.

By embracing benchmark-driven formative testing, the field can conduct more rigorous early-stage research that provides a robust foundation for subsequent confirmatory trials. Ultimately, to improve the quality and efficiency of the entire intervention research-to-development pipeline, such early-stage work should systematically and iteratively assess DTx using real-world RWE. The DTx RWE Framework recently proposed by Kim and colleagues [8] incorporates RWE-guided decision-making against benchmarks congruent with existing regulatory standards throughout the DTx lifecycle. The Framework’s step-by-step process aims to streamline the research and development-regulatory testing-public release process, facilitate greater transparency, and produce DTx that reflect current best practices and are more readily translatable to real-life settings. This has important public health implications, as emerging DTx are poised to collectively reduce health care burden and expand access to high-quality care via tailoring algorithms that deliver personalized and precise multicomponent treatment [9]. This study applied principles from the DTx RWE Framework in conducting benchmark-driven formative testing of an early-stage DTx intervention to enhance affective response during physical activity (PA) among physically inactive adults with overweight or obesity.

### This Study

#### Overview

Inadequate PA engagement (ie, <150 min/week of moderate intensity physical activity [MPA]) confers substantial risk for a variety of chronic diseases, including cancer [10]. Despite widespread public health initiatives—and many individuals reporting a desire to become more physically active—less than 5% of adults actually meet the ≥150 minute/week MPA threshold [11]. Interventions targeting social-cognitive factors (eg, self-efficacy, intentions, and beliefs) have had limited success, accounting for only 31% of the variability in PA engagement [12-15]. This PA intention-behavior gap suggests that noncognitive factors might be hijacking people’s best intentions to engage in PA [16]. According to the theory of hedonic motivation, humans are naturally drawn to activities that are pleasurable and avoid unpleasant activities [17]. Previous affective responses to a given behavior inform the development of affectively charged motivations to pursue or avoid the behavior in the future [18]. People with overweight or obesity are more likely than individuals of a healthy weight to experience pronounced negative affect (eg, discomfort) while engaging in PA, therefore decreasing the likelihood they will consistently engage in PA [19].

Factors that are potentially readily modifiable, such as PA intensity, type, and context, have been shown to be significant predictors of the affective response during PA. For example, most people experience pleasure when engaging in low-intensity PA and displeasure while engaging in vigorous-intensity PA [20], but affective responses to MPA are heterogeneous [21]. At moderate intensities, affective responses might be determined by the type (eg, hiking and dancing) or context (eg, being with others and outdoors) of PA [22-25]. Ultimately, affective responses vary inter- and intrapersonally and impact future PA behavior—but whether they can be experimentally manipulated to promote PA engagement in the real world remains unknown [26,27].

#### Developing a DTx Solution

Therefore, an existing DTx called MyDayPlan was modified to experimentally manipulate affective mechanisms associated with MPA among physically inactive adults with overweight or obesity. MyDayPlan has previously increased PA engagement in similar populations by leveraging self-regulation strategies posed by the Health Action Process Approach [28]. Specifically, smartphone sessions provide daily PA goals and prompt participants to plan how, where, and when they will accomplish the goal (action planning), identify potential barriers and solutions (coping planning), and monitor their goal progress (self-monitoring) [29]. In its original form, MyDayPlan provided goals focused on sustaining a certain intensity of PA (ie, “intensity-based goals”). The eMOTION Study incorporated affect-based PA goals and enhancements to modify affective mechanisms related to PA into MyDayPlan’s existing self-regulatory framework. New affect-based goals asked participants to engage in PA types and contexts that would allow them to experience positive affect (eg, enjoyment) during PA. We hypothesized that participants who received affect-based goals would report more positive affective experiences during PA than participants who received intensity-based goals, which asked participants to attain a target heart rate. Iterative internal testing among research team members was performed to achieve a minimum viable product with a functioning interface, error-free programming, and a reasonable time burden.

#### Benchmark-Driven Formative Testing

Next, following guidance from Phase II of the DTx RWE framework, SMART (specific, measurable, actionable, realistic, and timely [or time-bound]) benchmarks were defined (Textbox 1). Benchmarks required the DTx to reach safety, plausibility, accessibility, usability, sustainability, feasibility, and equity standards during a formative study before initiating a resource-intensive Comparison Study to test efficacy. Broadly, benchmarks ensured that the DTx was unlikely to cause harm (ie, safety); the affect-based goals were associated with greater PA enjoyment compared to the intensity-based control (ie, plausibility); and the DTx was easy to use (ie, accessibility and usability); and practical for study staff and participants to sustain over time (ie, feasibility and sustainability). We also explored whether DTx plausibility and accessibility were approximately equal for all participants, regardless of individual-level traits (ie, equity; this benchmark was exploratory and not statistically powered). This paper summarizes results from benchmark-driven formative testing of the eMOTION DTx by measuring performance against these a priori established benchmarks.

**eMOTION Formative Study SMART (specific, measurable, actionable, realistic, and timely [or time-bound]) benchmarks.**

Safety:

- Only <1% of Formative Study participants will experience an adverse event.

Plausibility:

- The majority of participants (ie, ≥51%) receiving affect-based goals will report a perceived increase in enjoyment of physical activity at the end of the study.

Accessibility and usability:

- The daily intervention sessions and Fitbit smartwatch features will receive an average score at or above the System Usability Scale cutoff point (≥68).
- The majority of participants (ie, ≥51%) will report that they are able to (1) read the wording of the survey questions, (2) understand the wording of the survey questions, (3) select their answers for the survey questions, and (4) use the exercise settings on the Fitbit Versa smartwatch.
- The majority of participants (ie, ≥51%) will report being able to (1) read and (2) understand the daily intervention sessions on their phone.
- Participants will be given physical activity recommendations that appropriately consider their reported constraints.
- The majority of participants (ie, ≥51%) who remember receiving savoring questions will report being able to understand and follow them.

Sustainability and feasibility:

- The auto-detection algorithm for physical activity will correctly trigger event-contingent ecological momentary assessment (EMA) prompts ≥51% of the time.
- Less than 25% of participants will need to be sent a new Fitbit due to technical issues.
- A total of <70% of participants will report feeling dissatisfied with the Fitbit Versa (1) exercise settings, (2) prompt notifications, and (3) prompt burden.
- In addition, <25% of participants will be sent repeated (ie, >1) reminders to open their Fitbit app and sync their study data.
- Moreover, <70% of participants who remember completing daily intervention sessions will report feeling dissatisfied with the daily intervention sessions.
- Additionally, <70% of participants will report feeling dissatisfied with the Fitbit app.
- In total, <70% of participants who remember receiving physical activity recommendations will report feeling dissatisfied with the physical activity recommendations.
- Furthermore, <70% of participants who remember receiving savoring questions will report feeling dissatisfied with the savoring questions.

Equity:

- Plausibility and accessibility of the eMOTION Intervention will be approximately equal between groups (ie, sex, race, ethnicity, age, BMI, income, mobility, and physical constraints).

## Methods

### Study Design

A 3-week formative study was used to test DTx RWE Framework benchmarks. Treatment components targeting intensity-based goals and affect-based goals were examined separately in different groups of participants. Stratified random assignment placed participants (N=36) into one of four groups (n=9 each): (1) intensity-based goals, (2) affect-based goals with the type and context enhancement, (3) affect-based goals with the savor enhancement, and (4) affect-based goals with type, context, and savor enhancements. Published recommendations for proof-of-concept and formative studies using similar measures to our own support the use of homogenous samples as small as 5 to 10 subjects per user group [30-32]. Our sample (n=9 per group) precludes comparisons via inferential statistics, but corresponds with current best practices by prioritizing efficient iterations central to early-stage work—and is ultimately still amenable to descriptive statistics to determine whether the DTx meets preliminary benchmarks. Strata within each condition were based on age (ie, <45 vs ≥45 years), sex at birth (ie, male vs female), and BMI classification (ie, those with overweight vs those with obesity) to allow for approximately equal numbers of subgroup participants within each treatment group. The study was triple-blinded so that participants, research staff interacting with participants, and statisticians conducting analyses were all unaware of group assignment. Methods were preregistered in Open Science Framework [7].

### Participants

Rolling recruitment occurred from September 2023 to March 2024 via ResearchMatch, a national health volunteer registry created by academic institutions and supported by the National Institutes of Health as part of the Clinical Translational Science Award program. An online screener assessed participant eligibility. The eligibility criteria are shown in Textbox 2.

**Eligibility criteria.**

Inclusion criteria were as follows:

- Ages 18 years and older.
- Residing in the United States.
- BMI ≥ 25 kg/m^2^.
- Current engagement in ≤60 minutes of structured physical activity (PA) per week.
- Smartphone ownership.
- Residing in an area with Wi-Fi connectivity.
- Able to speak and read English.
- Interested in starting a PA program.
- Willing to wear a smartwatch every day.
- Able to read the small font on a smartwatch screen.

Exclusion criteria screened out individuals who:

- Had cognitive disabilities precluding participation and/or the ability to provide informed consent.
- Had physical disabilities or medical issues limiting PA engagement.
- Were unable or unwilling to wear a smartwatch.
- Were currently pregnant.

### Ethical Considerations

All procedures were conducted remotely, and approval was provided by the University of Southern California Institutional Review Board (ID UP-22-00332). Eligible individuals provided written informed consent to participate in study procedures. Data were not deidentified or anonymized, so a number of protections were implemented to maintain participant privacy and confidentiality. These included the use of pseudonyms in place of names to organize study records, as well as the storage of data on secure databases and encrypted university servers. Participants were compensated up to US $75 for completing all study procedures.

### Procedures

Following informed consent, participants completed a baseline questionnaire via REDCap (Research Electronic Data Capture; Vanderbilt University) [33,34]. Onboarding included a 1- to 3-day trial that was “passed” by completing two brief text message surveys within a single day. Participants who “passed” the trial received a Fitbit Versa 3 smartwatch in the mail and attended a 45-minute orientation session to learn about participation activities, finish technological setup, and review a PA guidebook. For the first week of the study, assessment components (eg, Fitbit smartwatch monitoring and check-ins) were introduced gradually. Then, for the following 2 weeks, participants engaged in the full DTx. Participants were asked to wear the smartwatch on their nondominant wrist for ≥23 hours/day. Smartwatch-based ecological momentary assessment (EMA) prompts (ie, “check-ins”) were delivered during PA sessions to measure affective responses to PA. EMA is a methodology involving brief repeated surveys that capture subjective experiences as they unfold in real time [35]. Participants were also instructed to press an exercise button on the smartwatch before starting a planned PA session. Researchers performed remote monitoring of participant compliance and contacted participants if missing data were detected in the study server. Finally, an exit interview and poststudy questionnaire collected feedback relating to the formative study’s SMART benchmarks.

### DTx Intervention

The eMOTION DTx was a multicomponent treatment package that was delivered under unsupervised, real-world settings and included the following elements.

#### PA Guidance and Scheduling

Participants were given a prescription to start at their own pace and comfort level, slowly increasing MPA volume over the course of the study. Because PA was performed asynchronously, a digital guidebook was provided as a reference summarizing strategies to gauge PA intensity and proper warm-up and cool-down procedures. Additionally, on Sundays, participants completed a smartphone-based scheduling survey by indicating the days they intended to engage in PA for that upcoming week.

#### Daily PA Goal Sessions

On days participants reported they intended to engage in PA, they completed 2 smartphone-based interactive intervention sessions: one in the morning and one in the evening. A text message push notification and up to 4 reminder notifications (once per hour) with a REDCap link alerted participants to complete each session. Morning sessions were scheduled for 6 AM local time. They provided a PA goal for the day (“goal module”) that differed according to the participant’s random group assignment. Participants then created a plan (“action plan module”) to achieve their goal by specifying details, including the time they planned to engage in PA that day. The final module (“coping plan module”) asked participants to anticipate barriers and brainstorm potential solutions to reach their goal. Text messages for evening sessions were sent at 7 PM local time and featured a “self-monitoring module” that asked participants to reflect on whether they met their PA goal.

In the intensity-based goal condition, participants were asked to maintain a target heart rate during PA. Target heart rate was based on the approximate age-adjusted maximum heart rate (HRmax; HRmax = 207 – [0.7 × age]), reflecting 55% HRmax for week one and 60% HRmax for week two. In the affect-based goal condition, participants were asked to engage in a type (25% of daily goals) or context (75% of daily goals; alternating places, social situations, and listening contexts) of PA that they found enjoyable. The instructions were generic to encourage self-selection (eg, “Today, your goal is to try a TYPE of PA that makes the experience more enjoyable while you are doing it”).

#### Enhancements

Participants randomized to receive the type and context enhancement were provided tailored recommendations for PA types or contexts most likely to satisfy their unique psychological needs, as measured at baseline (ie, relatedness, social esteem, individual esteem, creativity, mindfulness, learning, challenge, entertainment, escapism, aesthetic appreciation, and morality). Recommendations were generated by an algorithm trained on data from a crowdsourced panel of adults on Amazon Mechanical Turk who were asked to rate a range of PA types and contexts on the extent to which they satisfied psychological needs. Further details on the algorithm are outside the scope of this paper. Recommendations suggested PA types or contexts matched to the participant’s most personally important psychological needs categories and eliminated types and contexts from consideration that coincided with any constraints reported at baseline.

Participants who were randomized to receive the SAVOR enhancement engaged in a brief savoring exercise to enhance the positive emotions experienced during their PA session. Immediately following the morning intervention session, these participants received a text message instructing them to click a REDCap link to complete the savoring exercise after they performed their planned PA session. The savoring exercise was comprised of two open-ended questions about the PA (eg, “Reflect on the most rewarding aspect of your PA session today. What made it feel like a success for you? How did this influence your experience?”) and surrounding environment (eg, “Let’s take a moment to focus on the environment in which you were active today. Can you describe what you saw, felt, heard, and smelled in that space? What aspects of the environment are you grateful for?”).

### Measures

#### Overview of Measures

Study variables evaluating DTx performance for SMART benchmarks were collected via questionnaires, videoconference exit interviews, and EMA. During exit interviews, if the participant’s initial response to an open-ended question was too vague, the surveyor followed up with neutrally worded clarifying questions. During response coding for analysis, if any response contained conflicting statements and true clarity could not be achieved, the response was always treated conservatively (ie, biased toward the null hypothesis). Measures are briefly summarized by benchmark type below and are elaborated in detail in Multimedia Appendix 1.

#### Safety

Safety was assessed by calculating the number of adverse events reported via official Institutional Review Board (IRB) channels, which allow participants and study teams to report events. Participants were given IRB contact information for this purpose during informed consent.

#### Plausibility

Plausibility was primarily assessed with the following exit interview question: “When comparing your experience with PA before and after participation in the eMOTION Study, do you personally feel as if the degree of enjoyment you feel while engaging in PA changed or stayed the same?” Responses were transcribed, and enjoyment was coded as “decreased” (0), “stayed the same” (1), or “increased” (2).

#### Accessibility and Usability

##### Daily Intervention Sessions and Fitbit Smartwatch

The poststudy questionnaire included the 10-item System Usability Scale (SUS) to assess the accessibility and usability of the daily intervention sessions and the Fitbit smartwatch [36]. Participants indicated the extent to which they agreed with each statement using a Likert Scale (“strongly disagree” {0} to “strongly agree” {4}). Negative items were reverse-scored, all items summed, and the sum multiplied by 2.5 to yield a total score ranging from 0 to 100. Items displayed acceptable reliability (Cronbach α=0.78 and 0.91 for daily goals and Fitbit smartwatch, respectively). Participants were also asked investigator-created questions in the exit interview about their ability to read and understand the wording of the daily intervention sessions and check-ins; select their answers for the check-ins; and use the exercise buttons on the smartwatch. Responses were transcribed and coded as “unable” (0) or “able” (1).

##### Tailored Physical Activity Recommendation Enhancement

Multiple measures assessed the accessibility and usability of type and context recommendations. During the exit interview, participants were asked whether they were “generally able to follow the PA [type and context] recommendations” they received. Responses were transcribed and coded as “unable” (0) or “able” (1). Reported inability was then considered alongside constraints reported at baseline. Specifically, in the baseline questionnaire, a set of questions asked about constraints related to PA type, location, and listening contexts. Participants indicated their ability to engage in 47 different activity types [37] by selecting as many options as applied for each one: “able to do it,” “physically unable to do it,” “no place to do it,” “no equipment to do it,” and “I don’t know what this is.” For some activities, “no equipment to do it” was not considered a constraint because no equipment was required (eg, bodyweight exercises); for others, “I don’t know what this is” did not apply because participants were provided with instructional videos for the activity. Next, participants indicated their ability to access 19 different locations and facilities for PA by selecting “I’m able” or “I don’t have access or can’t get there” for each. Finally, participants selected “yes” or “no” for whether they were able to listen to music, audiobooks, or other media on their smartphone during PA. The total number of possible constraints across PA types and contexts ranged from 0 to 68.

##### Savoring Enhancement

Participants were asked during the exit interview whether they were able to understand and follow the savoring exercises. Responses were transcribed and coded as “unable” (0) or “able” (1).

#### Sustainability and Feasibility

##### Satisfaction With DTx Components

Most benchmarks for sustainability and feasibility were assessed with the Delighted-Terrible Scale [38,39] in the poststudy questionnaire. Participants were asked to indicate how they felt about the smartwatch exercise settings; check-in notifications and burden; daily intervention sessions; smartphone data syncing procedures; and type, context, and/or savor enhancements, as applicable. Multiple choice options for each item were: “delighted” (8), “pleased” (7), “mostly satisfied” (6), “mixed- about equally satisfied and dissatisfied” (5), “mostly dissatisfied” (4), “unhappy” (3), “terrible” (2), “neutral- neither satisfied nor dissatisfied” (1), and “I never thought about it” (0). Response options “mostly dissatisfied,” “unhappy,” and “terrible” were considered as dissatisfaction with the given DTx component.

##### Fidelity of PA Auto-Detection Algorithm

An algorithm triggered check-ins when the smartwatch sensors detected PA engagement via sufficiently elevated 10-minute rolling average heart rate (ie, 55% to 60% of age-adjusted HRmax). Heart rate data from smartwatch sensors were considered alongside Fitabase software prompting records, which contained timestamps associated with the timing of smartwatch check-ins.

##### Research Staff Burden

Some measures assessed whether DTx-associated workloads were sustainable and feasible for research staff to maintain. A REDCap form was used to track smartwatch inventory and document technical difficulties requiring device replacement. Staff also recorded all instances where they had to remind participants to sync their smartphone data due to >48 hours elapsing without data reaching Fitabase servers.

##### Equity

To explore equity, the proportions of participants recalling increased PA enjoyment after the study, and rating the daily goals and smartwatch at or above the acceptable cutoff point (ie, SUS score ≥68.0), were compared across sex, race, ethnicity, age, BMI, income, mobility, and physical constraints subgroups (subjective comparisons, given a lack of statistical power). Subgroup data were collected with screening and baseline questionnaires. Sex (male or female), race (White or non-White), ethnicity (Hispanic or non-Hispanic), and income (unsure; ≤US $44,999; US $45,000-$84,999; US $85,000-$124,999; or ≥US $125,000) were directly assessed with single items. Age was calculated as the difference between date-of-birth and baseline questionnaire completion date, then split into quartiles (≤37 y, 38-43 y, 44-58 y, and ≥59 y). BMI was calculated using reported height and weight and coded according to established clinical cutoff points (those with overweight ≤29.9, those with obesity ≥30.0). Mobility was assessed with an investigator-developed measure, which asked participants to indicate the degree to which they agreed with 5 different statements (eg, “I have had trouble bending over”) using a 10-point sliding scale with the markers “not at all true” (1), “moderately true” (5), and “completely true” (10). Items were summed into a total mobility score (range 5 [least trouble] to 50 [most trouble]), which was then split into quartiles (≤13.5, 13.6-18.5, 18.6-28.5, and ≥28.6). Finally, for physical constraints, activity types designated as “physically unable to do it” by the participant at baseline were tallied to create a total number ranging from 0 to 47, which was split into tertiles (0, 1-4; ≥5).

#### Statistical Analysis

The formative study was not statistically powered to test hypotheses using inferential statistics. Rather, as is common for proof-of-concept trials, the sample size was chosen to balance monetary and personnel constraints while allowing the DTx to be applied and assessed in the target population. To minimize confirmation bias and reduce the risk of moving forward with a large-scale phase III comparison study prematurely, descriptive statistics were used to compare formative study evaluation data against a priori benchmarks with clear go or no-go criteria [8]. Sensitivity analyses featuring linear and logistic regression models were performed to compare person-level characteristics between the final sample and participants lost to follow-up. All analyses were performed using IBM SPSS Statistics software (version 29).

## Results

### Sample Characteristics

A total of 46 participants consented and enrolled in the eMOTION Formative Study. Of these, 9 participants left the study during onboarding and before the intervention: 2 withdrew due to personal obligations (ie, surgery and work demands), 1 was unenrolled due to technical difficulties associated with responding to text message EMA on their personal smartphone, and 6 were lost to follow-up without explanation (ie, study staff were unable to reestablish contact).

Table 1 provides baseline characteristics for the 37 participants who completed the intervention. This baseline sample was 46% female (n=17), 86% non-Hispanic (n=32), and 86% White (n=32). Mean age was 46.2 (SD 13.4) years. A broad range of household incomes was represented (Table 1). One additional participant in the sample was lost to follow-up after completing the intervention but before the poststudy questionnaire and exit interview. Therefore, a final analytic sample of 36 participants who completed the study and provided at least partial outcome data was retained. Sensitivity analyses confirmed that the final sample did not differ from participants lost to follow-up on the basis of their sex (*P*=.88), age (*P*=.82), or BMI (*P*=.98).

The baseline sample comprised 37 participants, while 36 participants provided at least some information for the main outcomes of interest by filling out the poststudy questionnaire and/or completing the exit interview.

**Table 1:** Participant characteristics assessed at baseline.

Characteristics		Values
Age (years), mean (SD)		46.2 (13.4)
**Sex, n (%)**		
	Male	17 (46)
	Female	20 (54)
**Ethnicity, n (%)**		
	Non-Hispanic	32 (86)
	Hispanic	5 (14)
**Race, n (%)**		
	White	32 (86)
	Black	4 (11)
	Asian	1 (3)
	Native American	0 (0)
	Pacific Islander	0 (0)
	Multiracial	0 (0)
**Household income (US $), n (%)**		
	≤44,999	7 (19)
	45,000 to 84,999	9 (24)
	85,000 to 124,999	8 (22)
	≥125,000	10 (27)
	Unsure	3 (8)

### SMART Benchmarks

#### Safety

No adverse events were reported during the study. With 0% of participants experiencing an adverse event, the safety benchmark (<1% of participants) was met.

#### Plausibility

Of the 27 participants who received affect-based goals, 14 (52%) reported during the exit interview that PA enjoyment increased over the course of the study (ie, comparing enjoyment before the study vs after the study), while 13 (48%) said enjoyment stayed the same. Therefore, the plausibility benchmark was just met (ie, ≥51% recalled an overall increase in enjoyment).

#### Accessibility and Usability

##### Daily Intervention Sessions and Fitbit Smartwatch

The daily intervention sessions and Fitbit smartwatch received average total SUS scores of 72.4 (SD 17.2) and 73.1 (SD 22.9), respectively. These scores exceeded the prespecified cutoff point in the SMART benchmarks (ie, ≥68). Post hoc calculations additionally determined that 23 (64%) and 26 (72%) participants rated the daily intervention sessions and Fitbit smartwatch components at-or-above the cutoff point (ie, ≥68). The majority of participants also reported being able to read the smartwatch check-ins (n=34, 97%) and daily intervention sessions (n=34, 97%), understand the check-ins (n=32, 91%) and daily sessions (n=34, 97%), select answers for check-ins (n=33, 94%), and use exercise settings on the smartwatch (n=27, 77%), therefore meeting the associated benchmarks.

##### Type and Context Enhancement

Although 18 participants received type and context recommendations, 3 did not remember receiving them. Table 2 depicts results from the 15 participants who received and remembered type and context recommendations. There was no discernable relationship between the number of activity constraints and reported ability to follow type and context recommendations, suggesting that the benchmark was met (ie, participants were given PA recommendations that appropriately considered their constraints).

Only data from participants who both received and remembered receiving PA type and context recommendations (ie, n=15) were included in the table. An additional 3 participants received, but did not remember receiving, recommendations; 5 participants incorrectly reported that they received type and context (ie, when they did not receive type and context); these data were excluded from the table.

**Table 2:** Accessibility of type and context recommendations.

PA^a^ constraint quartile	Reported being able to follow recommendations, n/N (%)
1	2/5 (40)
2	1/2 (50)
3	2/4 (50)
4	1/4 (25)

^a^PA: physical activity.

#### SAVOR Enhancement

Although 18 participants received savoring exercises, 4 did not remember receiving them. Of the 14 participants who received and remembered SAVOR, 12 (86%) reported being able to understand and follow the savoring exercises. Therefore, the associated benchmark (≥51% of participants who remember receiving savoring exercises will report being able to understand and follow them) was met.

#### Sustainability and Feasibility

##### Satisfaction With DTx Components

The proportion of participants reporting dissatisfaction with a DTx component (ie, Fitbit smartwatch exercise settings, check-in notifications, and check-in burden; daily intervention sessions; Fitbit app; PA type and context recommendations; and savoring exercises) ranged from 0% (n=0) to 39% (n=14; Table 3). Therefore, the associated benchmarks were met.

**Table 3:** Proportion of participants reporting dissatisfaction with digital therapeutics components.

Benchmark			Result, n (%)^a^
**<70% of participants will report feeling dissatisfied with Fitbit features**			
	Exercise settings	6 (17)	
	Check-in notifications	12 (33)	
	Check-in burden	14 (39)	
<70% of participants will report feeling dissatisfied with the Fitbit app			3 (8)
**<70% of participants who remember an intervention component will report feeling dissatisfied with it**			
	Daily intervention sessions^b^	3 (9)	
	PA recommendations^c^	3 (23)	
	Savoring exercises^d^	0 (0)	

^a^N=36 unless otherwise indicated.

^b^n=34 (2 participants did not remember receiving daily intervention sessions).

^c^n=13 (15 remembered receiving type and context recommendations, but 2 did not respond to the satisfaction item).

^d^n=12 (14 remembered receiving SAVOR, but 2 did not respond to the satisfaction item).

##### Fidelity of PA Auto-Detection Algorithm

All smartwatch check-ins were correctly sent when the rolling 10-minute average heart rate crossed the threshold specified in the algorithm (ie, 55%-60% HRmax). Therefore, the triggering algorithm achieved 100% accuracy and met the threshold (ie, the algorithm triggered smartwatch check-ins correctly ≥51% of the time).

##### Research Staff Burden

No replacement Fitbit smartwatches were sent out to participants due to technical issues; therefore, the meeting did not meet the associated benchmark (ie, <25% of participants needed a new Fitbit due to technical issues). Additionally, only 6/36 (ie, 17%) participants were sent a single reminder to sync their Fitbit data to their smartphone, and no participants (ie, 0%) required more than one reminder. Therefore, the benchmark (<25% of participants will be sent repeated [>1] reminders to open their Fitbit app and sync their study data) was met.

##### Equity

Results for the equity benchmark are summarized in Table 4. Values are the proportion of participants in each subgroup who reported increased PA enjoyment and who scored the daily goals and Fitbit smartwatch at or above the accessibility and usability cutoff point. Cell sizes were too small to use chi-square tests between groups. Subjective comparisons generally suggested equal proportions across group levels, with a few notable exceptions. First, plausibility differed by sex, with 64% (n=9) of women reporting increased enjoyment during the study compared to only 38% (n=5) of men. Additionally, non-White participants reported greater accessibility, usability (4/5 to 5/5, 80%-100% vs 19/31 to 21/31, 61%-68%), and plausibility (3/4, 75% vs 11/23, 48%) compared to White participants. Accessibility and usability were comparable across all income categories except for the highest income group, 90% (9/10) of whom rated the daily goals and Fitbit smartwatch at-or-above the cutoff point (compared to 3/6 and 4/8, 50% to 6/9, 69% in the other groups). Finally, accessibility, usability, and plausibility were lowest among individuals reporting the most physical constraints compared to those reporting fewer than 5 constraints or no constraints, as well as those reporting greater mobility impairment.

In Table 4, proportions and percentages for accessibility and usability reflect participants who scored the daily intervention sessions and Fitbit smartwatch, respectively, at-or-above the System Usability Scale cutoff of 68. Proportions and percentages in the plausibility column are the participants in the affect-based groups who reported increased PA enjoyment over the course of the study. A total of 36 completed the accessibility and usability items in the poststudy questionnaire, and 35 completed the plausibility item in the exit interview. Cell sizes were too small to conduct chi-Square comparisons, so potential group differences were subjectively assessed.

**Table 4:** Equity benchmark performance.

Characteristics			Accessibility and usability				Plausibility, n/N (%)
			Daily goals, n/N (%)		Fitbit, n/N (%)		
**Sex**							
	Male	11/17 (65)		13/17 (76)		5/13 (38)	
	Female	12/19 (63)		13/19 (68)		9/14 (64)	
**Ethnicity**							
	Non-Hispanic	21/32 (66)		24/32 (75)		13/25 (52)	
	Hispanic	2/4 (50)		2/4 (50)		1/2 (50)	
**Race**							
	White	19/31 (61)		21/31 (68)		11/23 (48)	
	Non-White	4/5 (80)		5/5 (100)		3/4 (75)	
**Household income (US $)**							
	≤44,999	3/6 (50)		3/6 (50)		1/5 (20)	
	45,000 to 84,999	6/9 (67)		6/9 (67)		7/7 (100)	
	85,000 to 124,999	4/8 (50)		5/8 (63)		3/7 (43)	
	≥125,000	9/10 (90)		9/10 (90)		3/5 (60)	
	Unsure	1/3 (33)		3/3 (100)		0/3 (0)	
**Age (years)**							
	≤37	3/9 (33)		6/9 (67)		4/7 (57)	
	38-43	6/9 (67)		6/9 (67)		2/7 (29)	
	44-58	8/9 (89)		8/9 (89)		4/5 (80)	
	≥59	6/9 (67)		6/9 (67)		4/8 (50)	
**BMI (kg/m^2^)**							
	Overweight	8/12 (67)		10/12 (83)		5/10 (50)	
	Obese	15/24 (63)		16/24 (67)		9/17 (53)	
**Mobility score**							
	≤13.5	6/9 (67)		7/9 (78)		5/7 (71)	
	13.6-18.5	6/9 (67)		8/9 (89)		3/6 (50)	
	18.6-28.5	6/9 (67)		7/9 (78)		4/8 (50)	
	≥28.6	5/9 (56)		4/9 (44)		2/6 (33)	
**Physical constraints**							
	No constraints	10/15 (67)		12/15 (80)		5/11 (45)	
	1-5 constraints	9/12 (75)		9/12 (75)		6/8 (75)	
	≥5 constraints	4/9 (44)		5/9 (56)		3/8 (38)	

## Discussion

### Principal Findings

The novel eMOTION DTx was systematically designed, developed, and formatively tested according to benchmarks recommended by the DTx RWE Framework. Multiple evidence-based intervention components were integrated to address the problem of inactivity due to low PA enjoyment among adults with overweight or obesity. Daily digitally interactive intervention sessions featured Health Action Process Approach self-regulatory strategies (ie, action planning, coping planning, and self-monitoring) to encourage attainment of PA goals [28]. Affect and Health Behavior Framework mechanisms, including affective responses during PA, were targeted with affect-based PA goals, as well as through two intervention enhancements [40]. The type and context enhancement involved a tailoring algorithm that generated recommendations for specific types and contexts of PA likely to satisfy personally relevant psychological needs. This was informed by a body of evidence demonstrating that the degree of importance ascribed to higher-order psychological needs varies between-persons [41-44], as well as by our preliminary work suggesting that various PA types and contexts differentially satisfy psychological needs and enhance positive affect during PA [45,46]. The SAVOR enhancement leveraged savoring exercises to intensify the influence of affective responses on PA [47,48]. Savoring is a mindfulness practice that enhances the saliency of positive experiences via attentional deployment and gratitude-making [49,50]. The DTx underwent rigorous internal testing and iterative refinements during phase I to arrive at a minimum viable product for application in phase II.

### Principal Results

SMART benchmarks determining success were defined a priori to maximize transparency and preregistered in accordance with open science best practices. Final benchmark results fully supported the safety and plausibility of the eMOTION DTx. Standards for safety and plausibility are considered the most important to uphold, with the Food and Drug Administration’s various approval routes all requiring new medical devices to demonstrate sufficient safety and efficacy before public release.

Findings also confirmed eMOTION DTx accessibility and usability. Accessibility and usability were assessed for multidevice (ie, smartphone and Fitbit smartwatch) user interface, study-related functions, and specific intervention content based on previous work, underscoring the importance of these areas for participant engagement and intervention delivery [51-53]. While objective sustainability and feasibility benchmarks were all met, minor real-time iterative adjustments were made to decrease smartwatch check-in burden in response to participant feedback. Early into formative testing, many participants reported receiving a high volume of check-ins throughout the day that were only meant to be received during PA sessions. To increase the specificity of the algorithm (ie, improve differentiation between low-intensity lifestyle activities and structured MPA sessions), the original heart rate threshold (ie, 10-minute rolling average ≥50% HRmax) was increased to 55% HRmax.

In an effort to increase the validity of accessibility, usability, sustainability, and feasibility feedback, participants were asked whether they remembered receiving each of the intervention components (ie, daily intervention sessions, PA recommendations, and savoring exercises). Terminology for these questions was consistent with that used during onboarding and throughout all study materials. Only those who received and remembered receiving a given component were included in the respective analysis. Interestingly, we found that some participants had forgotten the intervention components they received during the study (ie, 2 did not remember receiving daily goal intervention sessions; 3, the tailored PA recommendations; and 4, the savoring exercises). This same general phenomenon has been described by other researchers engaging in complex, multicomponent interventions, in which participants fail to retrospectively identify—or otherwise misremember or misunderstand—critical aspects of the study [54,55]. By comparing objective study records with participant self-reports, we were able to control for this form of information bias [56].

Finally, equity in accessibility, usability, and plausibility was generally observed (via subjective comparisons) across sex, race, ethnicity, income, age, BMI, mobility, and physical constraints subgroups, although notable discrepancies did arise. First, a greater proportion of women reported increased PA enjoyment during the study compared to men. Previous work presents conflicting evidence for whether men or women are more likely to enjoy PA [57,58]. However, our findings might reflect those of others that consistently suggest the presence of sex-based differences in the relevance and plausibility of mHealth interventions for PA. Specifically, women are more likely to use PA-tracking mHealth apps [59], report using PA apps for the purpose of enjoyment [60], and experience more positive health outcomes following PA mHealth interventions compared to men [61]. DTx accessibility, usability, and plausibility also differed by race, but—contrary to what might be expected—favored non-White participants. This result is likely unreliable due to imbalanced cell sizes (ie, 31 White and 5 non-White) and was not necessarily a cause for concern, as the DTx was more accessible, usable, and efficacious for the traditionally disadvantaged group. Racial differences will be further explored using a larger sample in Phase III. More concerning trends were observed for household income, mobility, and physical constraint subgroups. Specifically, the DTx was most accessible and usable for the wealthiest and least efficacious for the lowest-income participants. The DTx was also the least accessible, usable, and least efficacious for participants with the most physical constraints and most limited mobility, respectively. To address these findings, a free YouTube channel was created for participants. The channel included exercise videos using positive, upbeat formats and featuring diverse instructors, contexts, and PA types. Videos required only minimal space with no equipment necessary, and provided optional physical adaptations for each exercise. These changes sought to provide a larger number of enjoyable PA options that were convenient, accessible, and affordable to all participants, regardless of their income or physical abilities.

### Limitations

The extant literature contained very few validated measures for smartphone DTx accessibility, usability, sustainability, and feasibility. Therefore, we adapted existing measures from related technologies (eg, online websites and software systems) and created our own when appropriate. Investigator-developed items were highly specific to our particular DTx, potentially limiting generalizability to other DTx setups. Additionally, this phase II study’s small sample size precluded the use of inferential statistics; findings were descriptive, therefore, and should be interpreted with caution. Similarly, results for safety, plausibility, accessibility, usability, sustainability, feasibility, and equity of the eMOTION DTx reflect preliminary findings in our small, homogenous sample. Specific subgroups were at times collapsed into more general “affect-based” and “intensity-based” groups, and we were unable to explore whether specific combinations of enhancements drove the main effects. However, the phase III study will feature a sufficiently powered, diverse sample (N=280) to rigorously analyze within- and between-group differences in DTx efficacy and optimize treatment components.

### Conclusion

Overall, this study is one of the first to apply benchmarks recommended by the DTx RWE Framework to formatively test a novel digital intervention. By transparently reporting the specific measurement and analytic choices we made to operationalize the DTx RWE Framework, our study hopes to contribute methodology and results that can be replicated. This is an important undertaking, since recent investigations suggest that the inconsistent application of frameworks is hindering the advancement of knowledge in real-world implementation contexts [62]. Future work should aim to apply the DTx RWE Framework in other contexts to determine whether its processes are relevant across diverse health problems, target populations, and DTx formats.

All benchmarks were determined to be met satisfactorily following iterative improvements made to the eMOTION DTx during development. Preliminary evidence, therefore, supports a research transition into DTx RWE Framework phase III, in which a large-scale effectiveness trial will determine whether the eMOTION DTx produces a greater effect (ie, PA enjoyment) among the experimental (compared to control) group. Ultimately, our application of the DTx RWE Framework ensures that the design and development of the eMOTION DTx follow current best practices, increase the likelihood that regulatory standards are met, and facilitate an efficient translation from research to real-world practice.

## Appendix

Multimedia Appendix 1 Benchmark measurement and assessment details.

## Abbreviations

DTx: digital therapeutics
EMA: ecological momentary assessment
HRmax: maximum heart rate
IRB: Institutional Review Board
MCID: minimal clinically important difference
MPA: moderate intensity physical activity
ORBIT: Obesity-Related Behavioral Intervention Trials
PA: physical activity
REDCap: Research Electronic Data Capture
RWE: real-world evidence
SMART: specific, measurable, actionable, realistic, timely (or time-bound)
SUS: System Usability Scale

## Data Availability

The datasets generated and analyzed during this study are available from the corresponding author on reasonable request.
