# Supplementary material for: An Early-Stage Digital Therapeutic Intervention to Enhance Affective Response During Physical Activity Among Adults With Overweight or Obesity: Benchmark-Driven Formative Testing Study
Source: JMIR Hum Factors. 2026 Feb 20;13:e71472. doi: 10.2196/71472 (PMC12966821; doi:10.2196/71472)
Supplement: Multimedia Appendix 1 [file humanfactors_v13i1e71472_app1.docx]

**SUPPLEMENTAL FILE 1**

| **Safety** |
| --- |
| Less than 1% of Formative Study participants will experience an adverse event. |
| Measurement:  Official Institutional Review Board reporting channels (which allowed for both participants and the study team to report events) were used to calculate the number of reported adverse events. Participants were given IRB contact information for this purpose during Informed Consent. An adverse event was considered to be that which is unexpected, related (or possibly related) to the study procedures, and serious (i.e., adversely affecting the relationship between risks and benefits of participation in a serious manner, such as requiring hospitalization, causing persistent/significant disability, jeopardizing health, or requiring surgical or medical intervention to avoid persistent/significant disability or death). This included medical situations requiring follow-up after urgent care with a specialist. The adverse event must have had at least reasonable possibility of being caused by the study procedures, and not solely by a subject's underlying conditions or risk factor profile. |
| Benchmark Performance Assessment:  The total number of participants experiencing an adverse event was divided by the total number of eMOTION participants. |

| **Plausibility** |
| --- |
| The majority of participants (i.e., 51%) receiving affect-based goals will report a perceived increase in enjoyment of physical activity at the end of the study. |
| Measurement:  During the exit interview, participants were asked, "When comparing your experience with physical activity before and after participation in the eMOTION Study… Do you personally feel as if the degree of enjoyment you feel while engaging in physical activity changed or stayed the same?". Only those who were randomized to receive the affect-based goals were included in subsequent calculations. Responses were transcribed. If the participant’s initial response was too vague and directionality of any change was not explicitly mentioned, the surveyor followed up with additional questions to better understand (e.g., “you say it changed, but can you tell me in what way?” or “could you clarify the direction of that change you mentioned?”). If responses were mixed (i.e., containing conflicting statements about enjoyment) and true clarity was not achieved, then the response was conservatively considered to indicate “no change.” An example scenario which would have been coded as “no change” is if the participant began by stating there was no change in enjoyment, but then stated that they found themselves enjoying the activity more than before—but feeling annoyed with study requirements. |
| Benchmark Performance Assessment:  The number of “affect-based” participants who indicated a positive change in enjoyment (i.e., enjoyment increased) were divided by the total number of “affect-based” participants. |

| **Accessibility/Usability** | | | |  |
| --- | --- | --- | --- | --- |
| *Daily Intervention Sessions & Fitbit Smartwatch* | | | |  |
|  | 1. The daily intervention sessions and Fitbit smartwatch features will receive an average score at or above the System Usability Scale cutpoint (≥ 68). | | |  |
|  | | Measurement:  The Post-Study Questionnaire asked participants to fill out two adapted versions of the System Usability Scale (SUS).^1^ The first measure asked questions about daily intervention sessions, while the second asked about Fitbit smartwatch features. For each measure, participants used a 5-point Likert Scale to indicate the degree to which they agreed with 10 statements | |  |
|  | | | (responses ranged from “strongly disagree” [1] to “strongly agree” [5]). The adapted SUS is below, with wording variations for the daily intervention sessions (first phrase in brackets) and Fitbit smartwatch features (second phrase in brackets) included:   1. I think that I would like to [do the daily intervention sessions/use the Fitbit smartwatch features] in the future. 2. I found the [daily intervention sessions/Fitbit smartwatch features] unnecessarily complex. 3. I thought the [daily intervention sessions/Fitbit smartwatch features] were easy to [do/use]. 4. I think I would need the support of a technical person to be able to [do the daily intervention sessions/use the Fitbit smartwatch features]. 5. I found the various [parts of the daily intervention sessions/functions in the Fitbit smartwatch] were well integrated. 6. I thought there was too much inconsistency in the [daily intervention sessions/Fitbit smartwatch features]. 7. I would imagine that most people would learn to [do the daily intervention sessions/use the Fitbit smartwatch features] very quickly. 8. I found the [daily intervention sessions/Fitbit smartwatch features] very cumbersome to [do/use]. 9. I felt very confident [doing the daily intervention sessions/using the Fitbit smartwatch features]. 10. I needed to learn a lot of things before I could get going with the [daily intervention sessions/Fitbit smartwatch features].   For the daily intervention sessions SUS, participants were instructed, “Please tell us what you thought about the Fitbit smartwatch features. These refer to receiving notifications on your Fitbit, answering prompts on your Fitbit, and using your Fitbit during physical activity. Score the following 10-items on a 5-point Likert scale ranging from "strongly disagree" (1) to "strongly agree" (5).” For the Fitbit smartwatch features SUS, participants were instructed, “Please tell us what you thought about the daily physical activity intervention sessions. This refers to the text messages on your smartphone in the morning and evening with links to an online form to complete about your physical activity goals for that day. Score the following 10-items on a 5-point Likert scale ranging from "strongly disagree" (1) to "strongly agree" (5).” |  |
|  | | | Benchmark Performance Assessment:  To calculate the SUS score, score contributions from each item were summed. Each item's score contribution ranged from 0 to 4. For items 1,3,5,7,and 9 the score contribution was treated as the scale position (i.e., 1, 2, 3, 4, or 5) minus 1. For items 2,4,6,8 and 10, the contribution was 5 minus the scale position (i.e., 1, 2, 3, 4, or 5). The sum of the scores was then multiplied by 2.5 to obtain the overall value of system usability. This was performed for both the daily intervention session and Fitbit smartwatch feature SUS versions. |  |
|  | 1. The majority of participants (i.e., ≥51%) will report that they were able to (a) read the wording of the survey questions; (b) understand the wording of the survey questions; (c) select their answers for the survey questions; and (d) use the exercise settings on the Fitbit Versa smartwatch. | | |  |
|  | | Measurement:  During the exit interview, participants were asked a series of yes/no questions. These were as follows.   1. Were you able to read the wording of the survey questions on your Fitbit smartwatch? 2. Were you able to understand the wording of the survey questions on your Fitbit smartwatch? 3. Were you able to select your answers for the survey questions on your Fitbit smartwatch? | |  |
|  | | 1. Were you able to use the exercise settings on your Fitbit smartwatch? These include pressing the “start” and “end” buttons and viewing your heart rate during physical activity.   Responses to these questions were transcribed. If participants began a response by saying, “no, but….” and proceeded to list something that indicated they did have trouble, the response was conservatively considered to be “yes,” and feedback provided was noted by the research team. If the participant answered “yes,” the team member followed up with the question, “If not, what was the issue?” to collect feedback on how to improve the Fitbit interface. | | |
|  | | Benchmark Performance Assessment:  For each of the four areas/questions, the number of participants responding “yes” were divided by the total number of participants to calculate the performance toward the benchmark. | | |
|  | 1. The majority of participants (i.e., ≥51%) will report being able to (a) read and (b) understand the daily intervention sessions on their phone. | | | |
|  | | Measurement:  During the exit interview, participants were asked two yes/no questions. These were as follows.   1. Were you able to read the daily intervention sessions on your phone? 2. Were you able to understand the daily intervention sessions on your phone?   Responses to these questions were transcribed. If participants began a response by saying, “no, but….” and proceeded to list something that indicated they did have trouble, the response was conservatively considered to be “yes,” and feedback provided was noted by the research team. If the participant answered “yes,” the team member followed up with the question, “If not, what was the issue?” to collect feedback on how to improve the smartphone interface. | | |
|  | | Benchmark Performance Assessment:  The number of participants responding “yes” were divided by the total number of participants for each of the two categories to calculate performance toward the benchmark. | | |
| *TYPE/CONTEXT Enhancement* | | | | |
|  | Participants were given physical activity recommendations that appropriately consider their reported constraints. | | | |
|  | Measurement:  To meet this benchmark, the reported ability for participants to follow activity recommendations had to be similar across the full range of total activity constraints.  In the Baseline Questionnaire, a series of questions collected information on three categories of constraints: activity type, location, and listening context. Each category was assessed as below.   1. For activity type, participants were instructed, “Tell us whether you are able to do the following types of physical activity in the next 20 weeks. Choose all that apply.” This was followed by a matrix with 47 rows with different types of physical activity (e.g., volleyball, jump rope, gardening, swimming laps; taken from Ainsworth’s Physical Activity Compendium^2^), where participants indicated the following for each activity by checking the associated textbox: “able to do it,” “physically unable to do it,” “no place to do it,” “no equipment to do it,” “I don’t know what this is.” Participants were allowed to select as many checkboxes as applied for each activity type. The total number of physical activities with constraints were then tallied for each person. Any given activity was considered to be “constrained” if the participant indicated they were “physically unable to do it,” have “no place to do it,” have “no equipment to do it,” or that they “don’t know what this is.” For some activities, however, only “physically unable to do it” and “no place to do it” were considered to be valid constraints. For these activities, the option “no equipment to do it” did not apply because no equipment was required to perform the activity. Additionally, the option “I don’t know what this is” did not apply because the study team maintained a secure YouTube channel containing curated instructional videos led by trainers for each of | | | |
|  | these activity types. The YouTube channel was designed to feature a variety of exercises for which no equipment was needed so that participants who were unable to access special equipment (e.g., bicycle, soccer ball, roller skates, hockey stick) could follow along to an instructional exercise video from the comfort of their own home (or anywhere else). Participants were provided with the link to the YouTube channel in an email, and a team member also explained the YouTube channel’s purpose, as well as how to access/navigate it, during the Orientation Session. Therefore, the total possible constrained activities ranged from 0 (i.e., participant reported they were “able to do it” for all activity types) to 47 (i.e., every single activity was constrained).   1. Location constraints were collected with the question, “Tell us whether you are able to engage in physical activity at these locations/facilities in the next 20 weeks. Choose one.” These directions were followed by a list of 19 different locations (e.g., pool [indoors], parking lot, beach, trail, sidewalk, gym/health club); the response options for each were “I’m able” or “I don’t have access or can’t get there.” For each location, selecting “I don’t have access or can’t get there” was considered to be a constraint. The total possible location constraints ranged from 0 to 19. 2. For listening context constraints, participants were asked two yes/no questions: “Are you able to listen to something on an electronic device (e.g., phone, iPad, computer) while engaging in physical activity?” and “Are you able to access audio books on your phone?”. A “yes” response was considered to be a constraint; the total possible listening context constraints ranged from 0 to 2.   The number of constraints across all categories (activity type, location, and listening context) was then tallied to create a total constraint score ranging from 0 (no constraints) to 68 (maximum constraints).  Additionally, to assess whether activity recommendations provided to participants were appropriate, the Exit Interview first asked the following yes/no question: “You might have received daily physical activity goals that focused on enjoying your physical activity. An example prompt is, ‘Today your goal is to perform a physical activity that you enjoy and makes you feel good.’ Do you remember if you received these types of goals?”. If the participant answered “yes,” they were asked another yes/no question: “Do you remember receiving recommendations for the specific types of physical activity (like yoga or jogging), places to do physical activity (like in a park), people to do physical activity with (like a friend), or what to listen to during physical activity (like music)? These would have been in your daily intervention sessions in the mornings.” If the participant said “yes” again, a final follow-up yes/no question asked, "Were you generally able to follow the physical activity [type/context] recommendations you received?" The participant’s responses were transcribed. If participants began to respond to the last question by saying, “yes, but….” and proceeded to list something that indicated they did have trouble following the recommendations, the response was conservatively considered to be “no,” and feedback provided was noted by the research team. If the participant answered “no,” the team member followed up with the question, “If not, what was the issue?” to collect feedback on how to improve the recommendations. | | | |
|  | Benchmark Performance Assessment:  Cross-tabulation tables and a bar graph allowed for the reported ability to follow activity recommendations (yes/no) to be qualitatively compared across the full range of total constraints (activity type, location, and listening context combined) reported by participants. To achieve the benchmark, there could not be an observable trend between the number of constraints reported and a person's ability to follow the activity recommendations (e.g., when the graph was studied, participants in the upper half of the distribution for total constraints should be, at the very least, approximately equally as heterogenous as the lower half of the distribution for reported ability to engage [yes/no] in the activity recommendations). | | | |
| *SAVOR Enhancement* | | | | |
|  | The majority of participants (i.e., ≥51%) who remember receiving savoring questions will report being able to understand and follow them. | | | |
|  |  | | | |
|  | Measurement:  The Exit Interview first asked the following yes/no question: “You might have received daily physical activity goals that focused on enjoying your physical activity. An example prompt is, ‘Today your goal is to perform a physical activity that you enjoy and makes you feel good.’ Do you remember if you received these types of goals?”. If the participant answered “yes,” they were asked another yes/no question: “At the end of each day, some participants in this study received savoring questions asking them to recall things they enjoyed and think about the positive aspects of their physical activity that day. These questions were included in the evening physical activity intervention sessions that were completed on your smartphone. Do you remember if you received these types of questions?”. If they said “yes” again, a final follow-up yes/no question asked, “Were you generally able to understand and follow the savoring questions you received?" The participant’s responses were transcribed. If participants began responding to the last question by saying, “yes, but…” and proceeded to list something that indicated they were not able to understand and/or follow the savoring questions, the response was conservatively considered to be “no,” and feedback provided was noted by the research team. If the participant answered “no,” the team member followed up with the question, “If not, what was the issue?” to collect feedback on how to improve the recommendations. | | | |
|  | Benchmark Performance Assessment:  To calculate performance toward the benchmark, the number of participants responding “yes” to the question asking if they could understand and follow the savoring questions were divided by the total number of participants who remembered receiving savoring questions. | | | |

| **Sustainability/Feasibility** | | |  |
| --- | --- | --- | --- |
| *Satisfaction with DTx Components* | | |  |
|  | 1. <70% of participants will report feeling dissatisfied with the Fitbit Versa (a) exercise settings; (b) prompt notifications; and (c) prompt burden. | |  |
|  | | Measurement:  This benchmark was assessed with a series of three questions in the Post-Study Questionnaire. Each of these utilized the validated Delighted-Terrible Scale.^3^ Multiple-choice response options for each question were: “delighted” (8), “pleased” (7), “mostly satisfied” (6), “mixed- about equally satisfied and dissatisfied” (5), “mostly dissatisfied” (4), “unhappy” (3), “terrible” (2), “neutral- neither satisfied nor dissatisfied” (1), and “I never thought about it” (0). The three questions were as follows:   1. How do you feel about the exercise settings on your Fitbit smartwatch? These include pressing the "start" and "end" buttons and viewing your heart rate during physical activity. 2. How do you feel about the notifications you received to complete brief surveys on the smartwatch? These include feeling the haptic signal (vibration), the screen lighting up brightly enough and for enough time, the number of notifications you received, etc. 3. How do you feel about how often you received survey prompts on the smartwatch, and how long these surveys were? |  |
|  | | Benchmark Performance Assessment:  The number of participants who reported dissatisfaction (considered to be “mostly dissatisfied” [4], “unhappy” [3], and “terrible” [2]) were divided by the total number of participants for each of the questions separately to calculate percentages for this benchmark. |  |
|  | 1. <70% of participants who remember completing daily intervention sessions will report feeling dissatisfied with the daily intervention sessions. | |  |
|  | | Measurement:  For this benchmark, in the Post-Study Questionnaire, participants were asked the following yes/no question: “Do you remember completing daily intervention sessions in the morning and evening on your smartphone?”. If the participant answered “yes,” a follow-up question using the Delighted-Terrible Scale^3^ asked, “How do you feel about the daily intervention sessions?”. Multiple-choice response options for each Delighted-Terrible Scale question were: “delighted” (8), “pleased” (7), “mostly satisfied” (6), “mixed- about equally satisfied and dissatisfied” (5), “mostly dissatisfied” (4), “unhappy” (3), “terrible” (2), “neutral- neither satisfied nor dissatisfied” (1), and “I never thought about it” (0). | |
|  | | Benchmark Performance Assessment:  To calculate performance toward this benchmark, the number of participants who reported dissatisfaction (considered to be “mostly dissatisfied” [4], “unhappy” [3], and “terrible” [2]) with the daily intervention sessions were divided by the total number of participants who remembered completing daily intervention sessions. | |
|  | 1. <70% of participants will report feeling dissatisfied with the Fitbit app. | | |
|  | | Measurement:  For this benchmark, in the Post-Study Questionnaire, all participants were asked the following Delighted-Terrible Scale^3^ question: “Our data collection system is designed to work through the Fitbit app on your smartphone. How do you feel about running the Fitbit app in the background to sync your data for this study?”. Multiple-choice response options for each Delighted-Terrible Scale question were: “delighted” (8), “pleased” (7), “mostly satisfied” (6), “mixed- about equally satisfied and dissatisfied” (5), “mostly dissatisfied” (4), “unhappy” (3), “terrible” (2), “neutral- neither satisfied nor dissatisfied” (1), and “I never thought about it” (0). | |
|  | | Benchmark Performance Assessment:  To calculate performance toward this benchmark, the number of participants who reported dissatisfaction (considered to be “mostly dissatisfied” [4], “unhappy” [3], and “terrible” [2]) with the Fitbit app were divided by the total number of participants. | |
|  | 1. <70% of participants who remember receiving physical activity recommendations will report feeling dissatisfied with the physical activity recommendations. | | |
|  | | Measurement:  In the Post-Study Questionnaire, participants were asked, “Do you remember receiving recommendations for the specific types (like yoga or jogging), places, or people (like with a friend or in a park) of physical activity you should perform? These would have been in your daily intervention sessions in the mornings.” If they responded with “yes,” then they completed an item that used the Delighted-Terrible Scale.^3^ This item asked, “How do you feel about the physical activity recommendations that you received?”. Multiple-choice response options were: “delighted” (8), “pleased” (7), “mostly satisfied” (6), “mixed- about equally satisfied and dissatisfied” (5), “mostly dissatisfied” (4), “unhappy” (3), “terrible” (2), “neutral- neither satisfied nor dissatisfied” (1), and “I never thought about it” (0). | |
|  | | Benchmark Performance Assessment:  To calculate performance toward this benchmark, the number of participants who reported dissatisfaction (considered to be “mostly dissatisfied” [4], “unhappy” [3], and “terrible” [2]) were divided by the total number of participants who remembered receiving activity recommendations. | |
|  | 1. <70% of participants who remember receiving savoring questions will report feeling dissatisfied with the savoring questions. | | |
|  | | Measurement:  In the Post-Study Questionnaire, participants were asked, “Do you remember if you received | |
|  | | savoring questions that asked you to recall how physical activity made you feel and take time to enjoy the positive feelings? These would have been part of your evening daily intervention session.” If they responded “yes,” then they completed an item that used the Delighted-Terrible Scale.^3^ This item asked, “How do you feel about the savoring questions?”. Multiple-choice response options were: “delighted” (8), “pleased” (7), “mostly satisfied” (6), “mixed- about equally satisfied and dissatisfied” (5), “mostly dissatisfied” (4), “unhappy” (3), “terrible” (2), “neutral- neither satisfied nor dissatisfied” (1), and “I never thought about it” (0). | |
|  | | Benchmark Performance Assessment:  To calculate performance toward this benchmark, the number of participants who reported dissatisfaction (considered to be “mostly dissatisfied” [4], “unhappy” [3], and “terrible” [2]) were divided by the total number of participants who remembered receiving savoring questions. | |
| *Fidelity of PA Auto-Detection Algorithm* | | | |
|  | The auto-detection algorithm for physical activity will correctly trigger event-contingent ecological momentary assessment (EMA) prompts ≥51% of the time. | | |
|  | Measurement:  This benchmark was assessed via data exports from Fitabase containing information about the rolling 10-min average heart rate (HR) leading up to an EMA prompt being triggered on the Fitbit smartwatch. The algorithm was set to trigger an EMA prompt when the 10-min rolling average HR hit ≥55% of the person's age-predicted HR max, but this % HRmax was adjusted as the study continued and we learned more about the threshold’s performance (the threshold was increased due to participant feedback indicating that we needed to improve the specificity of the prompting mechanism). | | |
|  | Benchmark Performance Assessment:  The number of correctly-triggered event-contingent EMA (i.e., the number of EMA sent when the rolling %HRmax is ≥ the threshold) were divided by the total number of event-contingent EMA sent during the study to calculate performance toward this benchmark. | | |
| *Research Staff Burden* | | | |
|  | 1. <25% of participants will need to be sent a new Fitbit due to technical issues. | | |
|  | | Measurement:  This benchmark was assessed via participant tracking procedures. Study staff kept records on REDCap to document the date a Fitbit device was sent out, the ID number for the device, and notes about whether a device had to be replaced due to technical difficulties or other reasons. | |
|  | | Benchmark Performance Assessment:  The number of participants who required a replacement device due to technical difficulties were divided by the total number of participants to determine performance toward this benchmark. | |
|  | 1. <25% of participants were sent repeated (i.e., >1) reminders to open their Fitbit app and sync their study data. | | |
|  | | Measurement:  This benchmark was assessed by records from study staff that cross-referenced syncing data from the Fitabase Engage platform and text message communications sent to participants via Google Voice. Staff reached out to participants to request that they sync their app when at least three 24-hour periods had passed without a data sync, according to the "last sync" variable in Fitabase Engage. | |
|  | | Benchmark Performance Assessment:  The number of participants requiring more than one reminder text was divided by the total number of participants. | |

| **Equity** |
| --- |
| Plausibility and accessibility of the eMOTION Intervention were approximately equal between groups: sex, race, ethnicity, age, BMI, income, mobility, and physical constraints. |
| Measurement:  While this could not be tested statistically due to a lack of power in the Formative Study to compare groups of subjects, to achieve the benchmark the intervention had to yield similar effects regardless of sex, race, ethnicity, age, BMI, income, mobility, and physical constraints. For plausibility, the proportion of participants reporting an increase in enjoyment after the study was compared across sub-groups. For accessibility, the proportion of participants rating DTx components above the standard SUS cutoff for sufficient usability (≥68) was compared across sub-groups. Sub-groups were measured as follows.   1. Sex was collected in the Screening Questionnaire with the following question: 2. What was your sex at birth? *Male, Female* 3. Race was collected with the following item in the Baseline Questionnaire: 4. Which one or more of the following would you say is your race?  *White, Black or African American, American Indian or Alaska Native, Asian Indian, Chinese, Filipino, Japanese, Korean, Vietnamese, Other Asian, Native Hawaiian, Guamanian or Chamorro, Samoan, Other Pacific Islander, Other (write in)* 5. Ethnicity was collected with the following item in the Baseline Questionnaire: 6. Are you Hispanic, Latino/a, or Spanish origin? *Yes; No *If yes* Mexican, Mexican American, Chicano/a; Puerto Rican; Cuban; Another Hispanic, Latino/a, or Spanish* 7. Age was calculated by taking the difference between the participant's birthdate and the day they complete the baseline questionnaire. Birthdate was collected in the Screening Questionnaire with the following question: 8. What is your date of birth?  *Response options: selection of date from digital calendar or free response (format mm-dd-yyyy)* 9. BMI was calculated from items collected in the Baseline Questionnaire with the following equation: weight [pounds] / (height [inches] + (height [feet]*12))^2 * 703. Items were as follows: 10. What is your current weight? *Response options: short text box accepting numbers between 50 and 700, accompanied by a (lbs) clarification label* 11. What is your height? *Response options: drop-down boxes to collect feet ("ft"; possible values were 4-8) and inches ("in"; possible values were 0-11) separately* 12. For income, participants were asked the following multiple-choice question in the Baseline Questionnaire: 13. Over the past 12 months, what was your annual HOUSEHOLD income before taxes (from all members of your household combined)?  *Less than $12,000; $12,000 to $24,999; $25,000 to $34,999; $35,000-$44,999; $45,000-$54,999; $55,000-$64,999; $65,000-$74,999; $75,000-$84,999; $85,000-$94,999; $95,000-$104,999; $105,000-$114,999; $115,000-$124,999; $125,000-$149,999; $150,000-$174,999; $175,000-$199,999; Greater than $200,000; Don’t know/Not sure* 14. Mobility was assessed in the Baseline Questionnaire with an investigator-created question set. Participants rated the degree to which they agreed with the following five statements relating to their mobility “in the past month (30 days)” using a 10-point sliding scale (*with markers “Not at all true” [1], “Moderately true” [5], “Completely true” [10]*): 15. I have had trouble bending over. 16. I have been tired or winded. 17. I have been unable to stand comfortably. 18. I have not been physically active. 19. I have been unable to walk far/quickly. |
| Scores for each mobility item were then tallied to create a total mobility score ranging from 5 (least trouble with mobility) to 50 (most trouble with mobility).   1. Physical constraints^a^ were assessed with a set of questions in the Baseline Questionnaire focused on constraints related to physical activity type. Participants were instructed, “Tell us whether you are able to do the following types of physical activity in the next 20 weeks. Choose all that apply.” This was followed by a matrix with 47 rows with different types of physical activity (e.g., volleyball, jump rope, gardening, swimming laps; taken from Ainsworth’s Physical Activity Compendium), where participants indicated the following for each activity by checking the associated textbox: “able to do it,” “physically unable to do it,” “no place to do it,” “no equipment to do it,” “I don’t know what this is.” Participants were allowed to select as many checkboxes as apply for each activity type. The total number “physically unable to do it” responses were tallied to create a final physical constraint score ranging from 0 (i.e., no physical constraints) to 47 (i.e., maximum physical constraints).   ^a^ The original analytic plan (published *a priori* on Open Science Framework) specified that the total number of reported activity type constraints (i.e., counting any of the following responses as indicative of a constraint: no place, no equipment, or physically unable to perform the activity) would be compared. However, we chose to tally only “physically unable to do it” responses, as we believe this makes more logical sense and improves equity comparisons based on physical constraints (rather than including contextual limitations, which aren’t directly indicative of physical ability). |
| Benchmark Performance Assessment:  Accessibility and plausibility of the intervention were compared between the following groups of participants: sex (male; female), race (white; non-white), ethnicity (Hispanic; non-Hispanic), age quartiles (≤37; 38-43; 44-58; ≥59)^b^, BMI (overweight [≤29.9]; obese [≥30.0])^b^, income (unsure; ≤$44,999; $45,000 to $84,999; $85,000 to $124,999; ≥$125,000), mobility quartiles (≤13.5; 13.6-18.5; 18.6-28.5; ≥28.6)^b^, and physical constraint tertiles (0; 1-4; ≥5)^b^. These between-group comparisons were a subjective examination of SUS scores (for accessibility) and reported change/lack of change in physical activity enjoyment (for plausibility) across all levels of each of the sub-groups. If these values were approximately equal between the groups, then the benchmark was achieved.  ^b^ The original analytic plan (published *a priori* on Open Science Framework) specified that age, BMI, mobility, and physical constraints would be treated as continuous variables (frequency distributions). However, post hoc data visualization indicated that it was necessary to transform these variables into quartiles, tertiles, and dichotomous variables due to heavily skewed frequency distributions and very small or empty cell sizes which would have precluded meaningful sub-group comparisons. |

**References**

1. Brooke J. SUS: A “Quick and Dirty” Usability Scale. In: Jordan PW, Thomas B, McClelland IL, Weerdmeester B, eds. *Usability Evaluation in Industry*. 1st ed. CRC Press; 1996:189-194.

2. Ainsworth BE, Haskell WL, Herrmann SD, et al. 2011 Compendium of Physical Activities: A Second Update of Codes and MET Values. *Med Sci Sports Exerc*. 2011;43(8):1575-1581. doi:10.1249/MSS.0b013e31821ece12

3. Westbrook RA. A rating scale for measuring product/service satisfaction. *J Mark*. 1980;44:68-72.
